# Comparing the Efficacy of a Mobile Phone-Based Blood Glucose Management System With Standard Clinic Care in Women With Gestational Diabetes: Randomized Controlled Trial

**DOI:** 10.2196/mhealth.9512

**Published:** 2018-03-20

**Authors:** Lucy Mackillop, Jane Elizabeth Hirst, Katy Jane Bartlett, Jacqueline Susan Birks, Lei Clifton, Andrew J Farmer, Oliver Gibson, Yvonne Kenworthy, Jonathan Cummings Levy, Lise Loerup, Oliver Rivero-Arias, Wai-Kit Ming, Carmelo Velardo, Lionel Tarassenko

**Affiliations:** ^1^ Oxford University Hospitals NHS Foundation Trust Headington United Kingdom; ^2^ Nuffield Department of Women's and Reproductive Health University of Oxford Oxford United Kingdom; ^3^ Centre for Statistics in Medicine University of Oxford Oxford United Kingdom; ^4^ Nuffield Department of Primary Care Health Sciences University of Oxford Oxford United Kingdom; ^5^ Institute of Biomedical Engineering University of Oxford Oxford United Kingdom; ^6^ Oxford Centre for Diabetes, Endocrinology and Metabolism Oxford University Hospitals NHS Foundation Trust Oxford United Kingdom; ^7^ National Perinatal Epidemiology Unit University Of Oxford Oxford United Kingdom; ^8^ Department of Obstetrics and Gynaecology Sun Yat-Sen University Guangzhou China

**Keywords:** gestational diabetes, pregnancy, digital health, blood glucose monitoring, app, GDM

## Abstract

**Background:**

Treatment of hyperglycemia in women with gestational diabetes mellitus (GDM) is associated with improved maternal and neonatal outcomes and requires intensive clinical input. This is currently achieved by hospital clinic attendance every 2 to 4 weeks with limited opportunity for intervention between these visits.

**Objective:**

We conducted a randomized controlled trial to determine whether the use of a mobile phone-based real-time blood glucose management system to manage women with GDM remotely was as effective in controlling blood glucose as standard care through clinic attendance.

**Methods:**

Women with an abnormal oral glucose tolerance test before 34 completed weeks of gestation were individually randomized to a mobile phone-based blood glucose management solution (GDm-health, the intervention) or routine clinic care. The primary outcome was change in mean blood glucose in each group from recruitment to delivery, calculated with adjustments made for number of blood glucose measurements, proportion of preprandial and postprandial readings, baseline characteristics, and length of time in the study.

**Results:**

A total of 203 women were randomized. Blood glucose data were available for 98 intervention and 85 control women. There was no significant difference in rate of change of blood glucose (–0.16 mmol/L in the intervention and –0.14 mmol/L in the control group per 28 days, *P*=.78). Women using the intervention had higher satisfaction with care (*P*=.049). Preterm birth was less common in the intervention group (5/101, 5.0% vs 13/102, 12.7%; OR 0.36, 95% CI 0.12-1.01). There were fewer cesarean deliveries compared with vaginal deliveries in the intervention group (27/101, 26.7% vs 47/102, 46.1%, *P*=.005). Other glycemic, maternal, and neonatal outcomes were similar in both groups. The median time from recruitment to delivery was similar (intervention: 54 days; control: 49 days; *P*=.23). However, there were significantly more blood glucose readings in the intervention group (mean 3.80 [SD 1.80] and mean 2.63 [SD 1.71] readings per day in the intervention and control groups, respectively; *P*<.001). There was no significant difference in direct health care costs between the two groups, with a mean cost difference of the intervention group compared to control of –£1044 (95% CI –£2186 to £99). There were no unexpected adverse outcomes.

**Conclusions:**

Remote blood glucocse monitoring in women with GDM is safe. We demonstrated superior data capture using GDm-health. Although glycemic control and maternal and neonatal outcomes were similar, women preferred this model of care. Further studies are required to explore whether digital health solutions can promote desired self-management lifestyle behaviors and dietetic adherence, and influence maternal and neonatal outcomes. Digital blood glucose monitoring may provide a scalable, practical method to address the growing burden of GDM around the world.

**Trial Registration:**

ClinicalTrials.gov NCT01916694; https://clinicaltrials.gov/ct2/show/NCT01916694 (Archived by WebCite at http://www.webcitation.org/6y3lh2BOQ)

## Introduction

The prevalence of gestational diabetes mellitus (GDM) is increasing, creating demand for sustainable, cost-effective, and innovative approaches to care. There is enthusiasm for integration of digital technologies into health systems [1]. Although there is some evidence of clinical benefit with the use of digital health technologies in type 1 and 2 diabetes [2-4], large studies are lacking in the GDM population. There is potential for an integrated digital health solution for GDM: the condition requires frequent self-monitoring of blood glucose over a short time frame (typically 3 months), pregnant women are a motivated group willing to engage with health monitoring and advice, and women of reproductive age usually have an excellent grasp of digital technologies. Combining a digital blood glucose diary with real-time clinician review and feedback may improve glycemic control, reduce diabetes-associated complications, and mean potentially fewer outpatient contacts with the diabetes care team with cost savings to the health system, and it is likely to be more acceptable to women.

Several groups have developed remote blood glucose monitoring systems for women with GDM; however, trials have been small in size with potentially significant sources of methodological bias [5]. In the context of these limitations, telehealth monitoring systems have not been demonstrated to be superior to standard care for glycemic control and clinical outcomes [6-9]. Limited evidence supports that digital solutions are acceptable to pregnant women and possibly reduce the number of clinic visits [7,9,10]. No trial to date has assessed the associated health care costs.

We developed a digital blood glucose management system, GDm-health, to facilitate remote blood glucose self-monitoring and bidirectional communication between the clinical team and pregnant women. The system, described in detail elsewhere [11], was developed by patients, midwives, obstetricians, physicians, and biomedical engineers, and showed high levels of user satisfaction in a small service evaluation project [12]. We hypothesized that the real-time feedback and support offered by the system would improve glycemic control in women with GDM. To test this hypothesis, we conducted a randomized trial to assess whether digital remote management of GDM improved glycemic control compared to standard paper-based blood glucose monitoring, with secondary outcomes of maternal and neonatal outcomes, cost of care, and patient satisfaction.

## Methods

### Study Design and Participants

This was a single-center, balanced randomization 1:1, open-label, parallel-group, individually randomized controlled trial, conducted in a large UK tertiary referral hospital between September 2013 and June 2015 [13].

### Sample Size

There were no published data on precise estimates of the likely standard deviation in mean blood glucose, making sample size calculation challenging. Therefore, we pragmatically decided to assume a standard deviation of 0.8 mmol/L for the mean blood glucose level at the end point. Thus, with 100 patients in each arm, we would be able to detect a difference between the arms of 0.32 mmol/L, with power of 80% and a significance level of .05.

### Allocation

Participants were randomly allocated to two groups: intervention (the GDm-health management system; Multimedia Appendix 1) or control (usual care). Randomization used a partial minimization procedure to balance important covariates including gestational age, weight, and ethnic group using the Oxford University Primary Care Clinical Trials Unit computer-generated randomization system Sortition [14].

This trial received ethical approval from National Research Ethics Service Committee South Central-Berkshire B (reference number 13/SC/0176). Written informed consent was obtained for each participant. The trial was registered at ClinicalTrials.gov (ref: NCT01916694).

### Participants

Screening for GDM was based on risk factors as per UK clinical guidelines [15] and GDM was defined using the International Association of Diabetes and Pregnancy Study Group [16] criteria.

Eligible women were aged between 18 and 45 years with a viable singleton pregnancy of less than 35 weeks and 0 days, and had GDM diagnosed by 75 g oral glucose tolerance test [13].

Following diagnosis of GDM, women were instructed to perform preprandial and 1-hour postprandial blood glucose monitoring and were given information about the trial. If after this initial week they did not require immediate treatment with insulin, they were eligible for inclusion.

All women were asked to test their blood glucose six times a day on at least 3 days of the week, as per the local guideline. This consisted of a fasting sample, 1-hour postbreakfast, prelunch, 1-hour postlunch, predinner, and 1-hour postdinner. The target blood glucose range was fasting readings ≥3.5 and ≤5.8 mmol/L and 1-hour postprandial readings less than 7.8 mmol/L. Thresholds for further dietetic support were the same in both groups. A decision to start pharmacological treatment was made by a number of doctors unblinded to treatment allocation, but following the same local treatment guidelines for participants in both the intervention and the control arms.

### Intervention and Control

#### Standard Clinic Care Group (Control Group)

Participants in the control group were instructed to record their blood glucose values in a paper diary. Every 2 to 4 weeks they attended the outpatient clinic for review. Women were instructed to contact the diabetes midwife if their blood glucose breached predefined thresholds [13].

#### Remote Glucose Monitoring Group (Intervention Group)

Participants in the intervention group were loaned a mobile phone with the preinstalled GDm-health app and taught how to record, tag, and review blood glucose readings by a research midwife. Every 4 to 8 weeks they attended the outpatient clinic (ie, half as many clinic visits as the standard clinic care group).

A diabetes midwife reviewed the blood glucose readings on a secure website at least three times a week. The system generated an alert if the same predefined thresholds as for the control group were breached [13]. An automatic alert was also generated if the participant was not recording a predefined number of blood glucose readings per week or more glucose testing strips were needed. A short message service (SMS) text message containing advice about diet, dose adjustments of hypoglycemic medications, and messages of encouragement were sent to the participant by the diabetes midwife between clinic visits via the website.

### Analysis of Blood Glucose Data

#### Primary Outcome

The predefined primary end point was the rate of change in glycemia, measured as a function of blood glucose measurements (mmol/L/28 days), compared between the two groups. Change over time in glycemia in both groups was compared from recruitment until delivery.

Timed and tagged blood glucose data were extracted from the GDm-health management system to determine glycemia for the intervention group. Blood glucose data for the control group were extracted from the paper diaries completed by the women at each clinic visit. Paper diary data were entered into an electronic file, with a subset double-entered to check accuracy.

Paper diaries were used in preference to glucose meter downloads because the meters employed (LifeScan OneTouch Ultra Mini) did not allow mealtime tagging. Meter-generated time stamps were found to be inadequate surrogates.

#### Secondary Outcomes

Other predefined markers of glycemia were rate of change of glycated hemoglobin A_1c_ (HbA_1c_); overall mean blood glucose and mean fasting, preprandial, and postprandial blood glucose; and time to treatment from recruitment in weeks. Maternal outcomes known to be associated with diabetic control were compared between the groups: weight was recorded at each visit and body mass index (BMI) was calculated, pregnancy-induced hypertension or preeclampsia, gestational age at delivery, birthweight and proportion of large for gestational age babies (>90th percentile for gestation and gender), mode of birth, and perineal severe trauma. Neonatal outcomes were shoulder dystocia or birth injury, neonatal hypoglycemia, neonatal hyperbilirubinemia, or admission to neonatal intensive care.

Participant attitudes were assessed using the Oxford Maternity Diabetes Treatment Satisfaction Questionnaire [12]. This 12-item questionnaire has previously been validated for this population and was given to all women who participated in the trial within 6 weeks of the birth of the baby. Questions 1 to 9 asked women about their satisfaction with their care, their relationship with their diabetes team, and the reliability and convenience of the system, and were scored on a 7-point Likert scale (0=not satisfied to 6=very satisfied). Question 10 asked women about whether they felt the number of visits was too few, just right, or too many; question 11 asked whether they would be interested in using a mobile phone app to help with blood glucose monitoring; and question 12 asked whether they would recommend the app to a family member or friend with GDM. Scores for the first nine questions were summed with a maximum score of 54.

Direct health care costs, within the UK National Health Service (NHS) were compared between the two groups from the time of recruitment until hospital discharge after birth of the mother and baby. The complete list of services included in the cost analysis is reported in Table A in Multimedia Appendix 2 [17-19]. It was assumed that for some clinical outcomes (eg, shoulder dystocia, birth trauma, or neonatal hypoglycemia), and to avoid double counting, the costs associated with these outcomes were captured by the hospital length of stay. The cost analysis aimed to identify the additional costs of one group versus the other; therefore, the costs of the glucose meter and strips were excluded from the analysis because these were recommended for identical use by women in both groups. As GDm-health is free to install on a participant’s mobile phone, no specific intervention costs were included in this analysis. We present unit costs, resource use, and costs separately between treatment arms [20]. Costs were expressed in 2014-2015 UK sterling pounds (£) and no discounting was employed given the short time horizon of the analysis [21].

### Statistical Analysis

The analyses were based on the intention-to-treat population, which included all patients randomized. The primary analysis of blood glucose was repeated for the per-protocol population. The inclusion criteria for the per-protocol analysis were the population with more than 67% of expected numbers of blood glucose measurements (at least 28 of 42 readings for weeks when on pharmacological treatment and at least 12 of 18 readings for weeks not on pharmacological treatment).

#### Primary Analysis

The primary objectives were to compare rate of change in glycemia in the intervention arm with that in the control arm. Glycemia was assessed as a function of blood glucose measurements. The dependent variable, the blood glucose measurement, was recorded by each patient up to six times per day between recruitment and delivery. The change in blood glucose over gestation was modeled using a linear regression equation. A random coefficient model was fitted that allowed for differences between patients in the rate of change of blood glucose. Factors included in the model as fixed effects were (1) a two-level factor indicating the treatment group; (2) a factor with three levels indicating the time of day of the blood glucose measurement, breakfast, lunch, or dinner; (c) a two-level factor indicating whether the measurement was premeal or postmeal; and (d) baseline characteristics.

#### Secondary Analyses

The methods of linear mixed models were used to analyze the HbA_1c_ data. The rate of change of HbA_1c_ over gestation was modeled using a first-order regression equation. A random coefficient model was fitted that allowed for differences between patients in the rate of change, as performed for the primary outcome.

To compare maternal and neonatal outcomes in the treatment groups, continuous normally distributed variables were analyzed using analysis of covariance, including baseline characteristics as covariates, and binary outcomes using logistic regression. Results are reported as a treatment effect or odds ratio with 95% confidence limits. For continuous variables that were not normally distributed, the median and interquartile ranges (IQRs) are reported and a nonparametric test was used to compare treatment groups. For binary variables with zero or very small number of events, exact logistic regression was used.

Costs were estimated by multiplying quantities of health care resource use by the corresponding unit costs (Multimedia Appendix 2, Table A). Descriptive statistics were employed to summarize health care resource use and costs between the two treatment arms. A complete-case analysis was carried out given the small number of missing data present in the dataset. Parametric mean cost differences and associated 95% confidence intervals for each category of resource use were calculated to identify potential cost differences [21]. In addition, a summary mean total cost per delivery over the trial period was computed adding the costs of antenatal care and intrapartum and postnatal care before discharge together.

All analyses were carried out using SAS version 9 (SAS Institute Inc, Cary, NC, USA) and Stata MP14 (StataCorp LP, College Station, TX, USA,).

## Results

### Participant Characteristics and Data Capture

**Figure 1:**
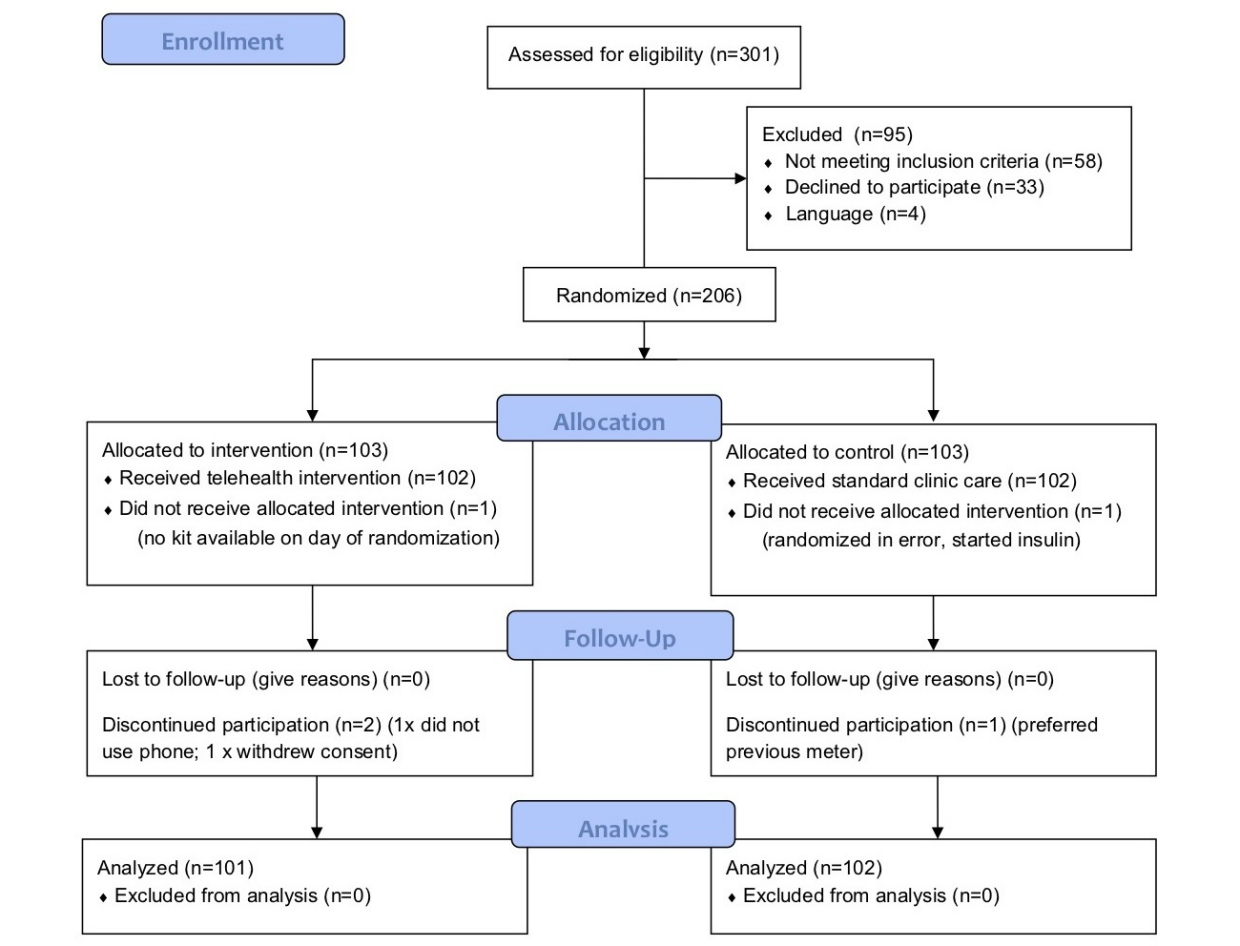
CONSORT Flow Diagram for TREAT GDM.

Of 301 women with GDM approached to participate in the study, 62 did not meet the inclusion criteria (6 outside age range, 22 had insulin prescribed after first week of monitoring, 11 were more than 34 weeks gestation, 6 had other medical conditions, 4 could not understand spoken English, and 13 other reasons) and 33 declined. Of the 206 women who met the inclusion criteria and provided written informed consent, 103 were randomized to the intervention group and 103 to the control. Two women in the intervention group and one in the control group chose to withdraw from the study before delivery, thus results from 101 women in the intervention and 102 in the control group are included in the intention-to-treat analysis (see Figure 1).

Baseline characteristics of the intervention and control groups were similar at recruitment (Table 1). At time of recruitment, 17 women in the intervention group and 13 women in the control group were taking hypoglycemic medication.

The number of hospital doctor visits were mean 4.65 (SD 2.89) and mean 5.06 (SD 2.86) in the intervention and control groups, respectively. The difference did not reach statistical significance.

Blood glucose data were obtained from 98 women (21,494 readings) for the intervention group; 85 patients (14,472 readings) in the control group used paper records. The median times from recruitment to delivery in the intervention and control groups were 54 (IQR 40 to 64) and 49 (IQR 41 to 60), respectively. Data capture was significantly greater in the intervention group with a mean 3.80 (SD 1.80) readings per day in the intervention group and mean 2.63 (SD 1.71) readings per day in the control group (*P*<.001).

Missing data in the intervention group were due to noncompliance with the protocol or technical failure. The possible reasons for missing data in the control group included noncompliance, missing blood glucose readings recorded by participants, or lost paper diaries.

In total, 78 women in the intervention group and 52 women in the control group were included in the per-protocol analysis.

### Primary Outcome

**Figure 2:**
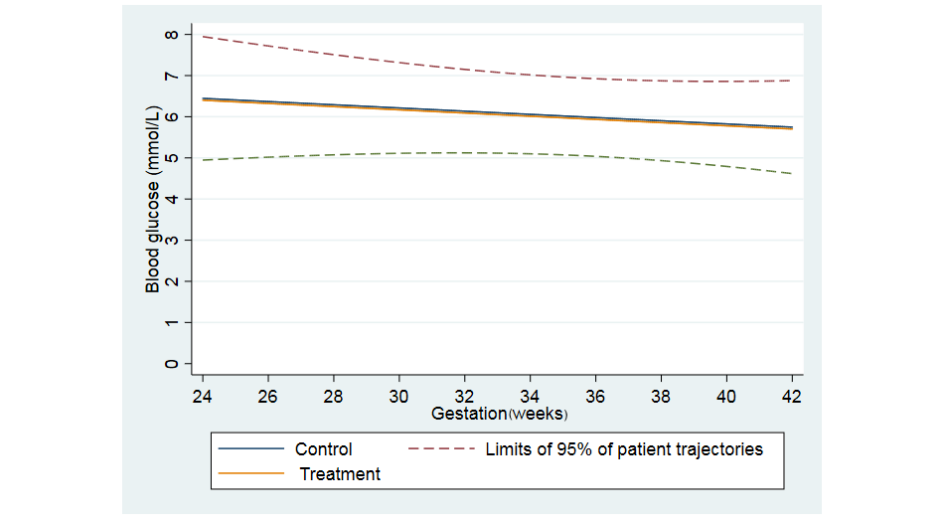
Change in mean blood glucose.

From study recruitment until delivery, the mean blood glucose fell in both groups. On average, blood glucose declined by 0.16 mmol/L/28 days in the intervention group and 0.14 mmol/L/28 days in the control group; the difference was not statistically significant (difference –0.01 mmol/L, 95% CI –0.10 to 0.08). Figure 2 shows change in mean blood glucose.

In the per-protocol analysis, the mean blood glucose decline was 0.17 mmol/L/28 days (95% CI –0.24 to –0.11) in the intervention group and 0.10 mmol/L/28 days (95% CI –0.19 to –0.01) in the control group. The difference between the two groups was not statistically significant.

### Secondary Outcomes

#### Glycemic Control

Data for HbA_1c_ levels were obtained from 100 women (320 HbA_1c_ values; mean 3.2 values per woman) in the intervention group and from 101 women (338 HbA_1c_ values; mean 3.4 values per woman) in the control group.

Despite an overall decrease in mean blood glucose, a marginal increase in HbA_1c_ was observed in both groups from recruitment until delivery, with a mean 0.02% rise per 28 days in the intervention group and a 0.03% rise per 28 days in the control group. There was no statistically significant difference (intervention vs control: –0.01%, 95% CI –0.05 to 0.03).

At delivery, 45 of 101 (44.6%) women in the intervention group and 57 of 102 (55.9%) women in the control group were on metformin (OR 0.63, 95% CI 0.36-1.10).

Mean blood glucose and range and percentage of “on target” readings over four weekly time points as a function of meal tags are presented in Tables B and C in Multimedia Appendix 2. Mixed model analysis showing the effect of BMI and smoking is presented in Table D in Multimedia Appendix 2.

Other predefined secondary outcomes, specifically, number of dose adjustments of hypoglycemic medications and maximum dose of insulin or metformin, were inconsistently recorded in the clinical record and have therefore not been included in the results.

#### Maternal and Neonatal Outcomes

Maternal and neonatal clinical outcomes are reported in Multimedia Appendix 3. Women in the intervention group had a median gestational age at delivery 3 days greater than those in the control group, but the difference was not statistically significant (log-rank test: χ^2^_1_=14.5, *P*=.22). Preterm birth was less common in the intervention group (5/101, 5.0%) versus in the control group (13/102, 12.7%; OR 0.36, 95% CI 0.12-1.01). The cesarean delivery rate compared with other modes of delivery was lower in the intervention group compared to the control group (27/101, 26.7% vs 47/102, 46.1%, *P*=.005), with notably fewer emergency cesarean deliveries in the intervention group. Rates of other maternal complications, including hypertensive disorders of pregnancy, perineal trauma, and maternal admission to a higher level of care, were low across both groups, with no significant differences demonstrated. Weight gain from recruitment to delivery did not differ between the groups.

**Table 1:** Baseline characteristics of participants (N=203). BMI: body mass index; GCSE: General Certificate of Secondary Education; GDM: gestational diabetes mellitus.

Characteristic		Intervention			Control		
		N^a^	Mean (SD)	n (%)	N^a^	Mean (SD)	n (%)
Maternal age (years)		101	33.9 (5.5)		102	33.0 (5.6)	
**Parity**		101			102		
	0			36 (35.6)			42 (41.2)
	1			33 (32.7)			40 (39.2)
	≥2			32 (31.7)			20 (19.6)
Height (m)		101	1.63 (0.08)		102	1.63 (0.07)	
Weight at booking (kg)		100	82.9 (18.2)		102	84.7 (21.5)	
BMI at booking (m/kg^2^)		100	31.1 (6.7)		102	31.6 (7.3)	
Smoking in pregnancy		101		3 (3.0)	102		5 (4.9)
Essential hypertension		101		2 (2.0)	101		6 (5.9)
First-degree relative with diabetes		99		39 (39.4)	100		43 (43.0)
Previous GDM^b^		65		10 (13.8)	60		7 (11.7)
Previous baby weighing >4.5 kg^b^		64		5 (7.8)	60		5 (8.3)
Previous cesarean delivery^b^		65		22 (33.8)	60		24 (40.0)
**Highest educational attainment**		101			99		
	GCSE or less			27 (26.7)			24 (24.2)
	A Level			22 (21.8)			30 (30.3)
	University			52 (51.5)			45 (45.5)
**Ethnic group**		100			102		
	White			77 (77.0)			80 (78.4)
	South Asian			10 (10.0)			13 (12.7)
	African/Caribbean			6 (6.0)			4 (3.9)
	East Asian			3 (3.0)			1 (1.0)
	Other			4 (4.0)			4 (3.9)
Gestational age at recruitment (weeks)		101	30.9 (3.6)		102	31.0 (3.4)	
**Oral glucose tolerance test (mmol/L)**							
	Fasting	98		5.2 (0.9)	96		5.2 (0.9)
	1 hour	79		9.9 (1.7)	87		10.4 (1.7)
	2 hour	99		7.4 (2.2)	97		7.0 (1.9)
Patients on metformin at recruitment		101		17 (16.8)	102		13 (12.7)
HbA_1c_^c^ at recruitment^d^ (%)		42	5.42 (0.34)		46	5.39 (0.35)	

^a^N refers to the total number of participants for whom data for each variable available.

^b^Multiparous women only.

^c^HbA_1c_: glycated hemoglobin A1c.

^d^HbA_1c_ at recruitment was measured between 18 and 35 weeks gestation.

Neonatal outcomes were similar between the groups. There was no significant difference observed between the groups with respect to mean birthweight, proportion of large for gestational age babies, neonatal sex, shoulder dystocia, neonatal hypoglycemia, neonatal jaundice, or admission to the neonatal intensive care unit.

#### Satisfaction with Care

In all, 60 of 102 patients in the control group and 60 of 101 patients in the intervention group returned the completed Oxford Maternity Diabetes Treatment Satisfaction Questionnaire. One question was misunderstood by approximately half of the patients and was omitted from the total score. Both groups reported high levels of satisfaction with the care they received (intervention: median 43, IQR 39-46; control: median 44.5, IQR 41-46; Kruskal-Wallis χ^2^_1_=3.9, *P*=.049). In the control group, 48 of 60 (80%), and 53 of 60 (88%) in the intervention group, felt that the number of visits was just right (*P*=.22) compared with too few or too many. In the intervention group, 57 of 60 women who used the app stated they would use it again and 51 of 60 in the control group said they would consider using a mobile phone app. In the intervention group, 59 of 60 women replied that they would recommend the app to friends or family with the same condition. Free-text comments emphasized the convenience of GDm-health, the additional support out of hospital, and the benefits of avoiding the hospital for appointments.

#### Cost Analysis

No statistically significant cost differences were observed between the two groups over the trial period (Multimedia Appendix 4). Estimated mean cost per delivery was £5697 (SD £3068) and £6741 (SD £4640) in the intervention and control groups, respectively, with a mean cost difference of –£1044 (95% CI –£2186 to £99).

#### Compliance With the Protocol

Compliance with blood glucose readings was significantly better in the intervention group. In all, 78 of 98 women in the intervention group and 52 of 85 women in the control group recorded at least 67% of the expected number of readings (OR 2.44, 95% CI 1.29-4.61).

## Discussion

### Principal Findings

In this randomized trial, digital remote management of blood glucose in women with GDM was associated with similar blood glucose control compared to usual care, as assessed by mean change in blood glucose. We demonstrated that women using the GDm-health system had significantly more blood glucose readings, higher satisfaction scores and fewer cesarean deliveries compared to women in the control group, and these reached statistical significance. There were longer gestations and fewer preterm births in the intervention group compared to the control group which did not reach statistical significance. Other maternal and neonatal outcomes were similar in both groups. We were not able to demonstrate a significant cost savings.

This digital health solution has proven popular with women who commented that they appreciated the additional support and monitoring it provided, as well as the perceived time and cost savings of avoiding hospital appointments. The UK strategy for improving health care is to give individuals shared access to their own health records and for them to be at the center of all decision making [1]. By directly allowing women to contribute to and access their blood glucose monitoring data, we believe GDm-health is a good example of a system moving toward this goal.

This is the largest randomized controlled trial to date of a digital health solution for the management of women with GDM. Our findings are in keeping with our published systematic review [5], which concluded there was no evidence of superior glycemic control using digital monitoring. As with other trials, patient satisfaction was higher in the intervention group. We did not show a significant effect on secondary clinical outcomes. It is of note that interventional trials in women with GDM that are powered to show significant effects on clinical endpoints are usually much larger than our trial (typically in excess of 500 women in each arm). These trials also compare any treatment against no treatment for GDM; therefore, they are likely to have a bigger effect on outcome [22].

Fewer women in the intervention group transitioned to hypoglycemic medication during the study, which may have been related to better dietetic adherence, although we did not collect data on diet and exercise in this study. Although not statistically significant, there was an average 3-day prolongation of pregnancy and fewer preterm births in the intervention group compared to the control group, which may have influenced the significantly fewer emergency cesarean deliveries. The clinical benefit of influencing self-managed lifestyle behaviors and dietetic adherence in this population using digital technologies warrants further study.

Determining the adequacy of blood glucose control during pregnancy is challenging. Although we report no difference in HbA_1c_, it is a poor measure of glucose control in the context of rapid changes in glycemia over a short period of time [23]. Likewise, we did not have access to continuous glucose monitoring, thus the overall linear rate of change of blood glucose we present was reliant on the woman’s capillary testing and may have missed differences in fetal glucose exposure during the trial. Assessing end organ effects such as fetal growth has been suggested as an indicator of blood glucose control [24]; however, by its nature this measure is retrospective indicating past rather than current glycemic status. Trends in fasting and preprandial and postprandial capillary blood glucose monitoring, therefore, remain the mainstay of glycemic assessment in women with GDM.

Capturing the data in the paper diaries presented several challenges in this trial, with incomplete, untagged, inaccurate, and missing records. Poor compliance has been associated with poor concordance between paper diaries and meter readings and poorer glycemic control [25]. Digital blood glucose recording with automated delivery of blood glucose data provides a reliable and secure source of data for clinical interpretation. Beyond fidelity in data capture, digital data linked to mealtimes and other clinical parameters (eg, fetal growth) could be used in dynamic analyses, giving feedback to patients and clinicians about overall trends in blood glucose control. In our service development cohort, we demonstrated that 2-week moving-average blood glucose values were significantly higher in women with GDM who delivered large for gestational age infants compared to those with normal infants [26]. For this trial, we did not incorporate any predictive algorithms into the system, other than a graphical display of blood glucose trends, as algorithms have yet to be validated for clinical practice. However, digital technologies incorporating artificial intelligence or clinician based feedback as well as optimized reporting and alerting visuals could have the potential to promote desired self-managed lifestyle behaviors and dietetic adherence.

Current thresholds for treatment targets in GDM are based on consensus, historical practice, and targets selected for use in clinical trials. The ability to accurately correlate dynamic data with clinical endpoints will be an important development for future research in GDM, eventually enabling individualized glycemic management plans. As technologies for continuous glucose monitoring become more reliable and affordable, a natural progression would be to incorporate their output into a digital health system such as GDm-health [27].

### Strengths and Limitations

The strengths of this study are the rigorous design and attention to randomization, which ensured similar groups at study entry and the high levels of follow-up until delivery in both groups. The trial was conducted under “real life” conditions in a busy maternity diabetes service. We considered a range of clinical and nonclinical outcomes, important for comprehensive evaluation of the potential benefits and harms of a new technology. We also present the first randomized comparison of direct costs of maternity-associated care.

The trial also has limitations. We were unable to demonstrate a difference in the number of clinic attendances between the groups, despite this being specified in the protocol. Booking follow-up appointments based on study allocation proved challenging, as routine 2-week, rather than modified 4-week follow-up appointments for participants assigned to receive the GDm-health intervention were often made by clerical staff, most of whom were unaware of the study. Therefore, it is not possible for us to determine whether this technology can safely replace clinic visits and this clearly impacted on direct health care costs.

A full economic assessment was not performed, with costs of implementation, medications, and indirect costs not included. The trial was conducted in a single referral center, where the technology was also developed and, as such, uptake and effectiveness may differ in other settings. We are currently evaluating a pilot scale-up program in three other public hospitals. At the time of writing, more than 250 women each month are using GDm-health, with similar satisfaction scores and sustained use over 2 years [28]. A further limitation is that the trial was limited to women who could understand written and spoken English to be able to provide valid consent without the need for an interpreter. In populations with large non-English speaking populations provision would need to be made to ensure equity of access to the system.

### Conclusion

There is a national drive to incorporate digital health solutions into routine UK health care delivered through the NHS; however, the evidence for their efficacy and clinical and cost-effectiveness is lacking. This pilot study describes the largest randomized evaluation to date of a system to monitor and manage GDM remotely. The system appears safe with comparable glycemic control, maternal, and newborn outcomes between allocated groups, with improved patient satisfaction and superior data capture in the intervention group. Further large, detailed health economic evaluation of these systems at scale is required to understand their potential impact on health care systems. Likewise, studies to understand whether such real-time digital monitoring systems incorporating continuous glucose monitoring technologies can provide new insights into predictive and bespoke individualized management plans are also required. Finally, studies to evaluate whether these digital systems have the potential to promote desired self-management behaviors and better dietetic adherence which could also influence clinical outcome, are required.

## Appendix

Multimedia Appendix 1 GDm-health app screenshots.

Multimedia Appendix 2 Supplementary Information.

Multimedia Appendix 3 Maternal and neonatal outcomes.

Multimedia Appendix 4 Breakdown of healthcare resource use and associated cost of intervention and control groups.

Multimedia Appendix 5 CONSORT-EHEALTH checklist (V 1.6.1).

## Abbreviations

BMI: body mass index
GDM: gestational diabetes mellitus
HbA _1c_: hemoglobin A1c
IQR: interquartile range
NHS: National Health Service
NIHR: National Institute for Health Research
