# Supplementary material for: Comparing the Efficacy of a Mobile Phone-Based Blood Glucose Management System With Standard Clinic Care in Women With Gestational Diabetes: Randomized Controlled Trial
Source: JMIR Mhealth Uhealth. 2018 Mar 20;6(3):e71. doi: 10.2196/mhealth.9512 (PMC5883074; doi:10.2196/mhealth.9512)
Supplement: Multimedia Appendix 1 [file mhealth_v6i3e71_app1.pptx]

## Slide 1
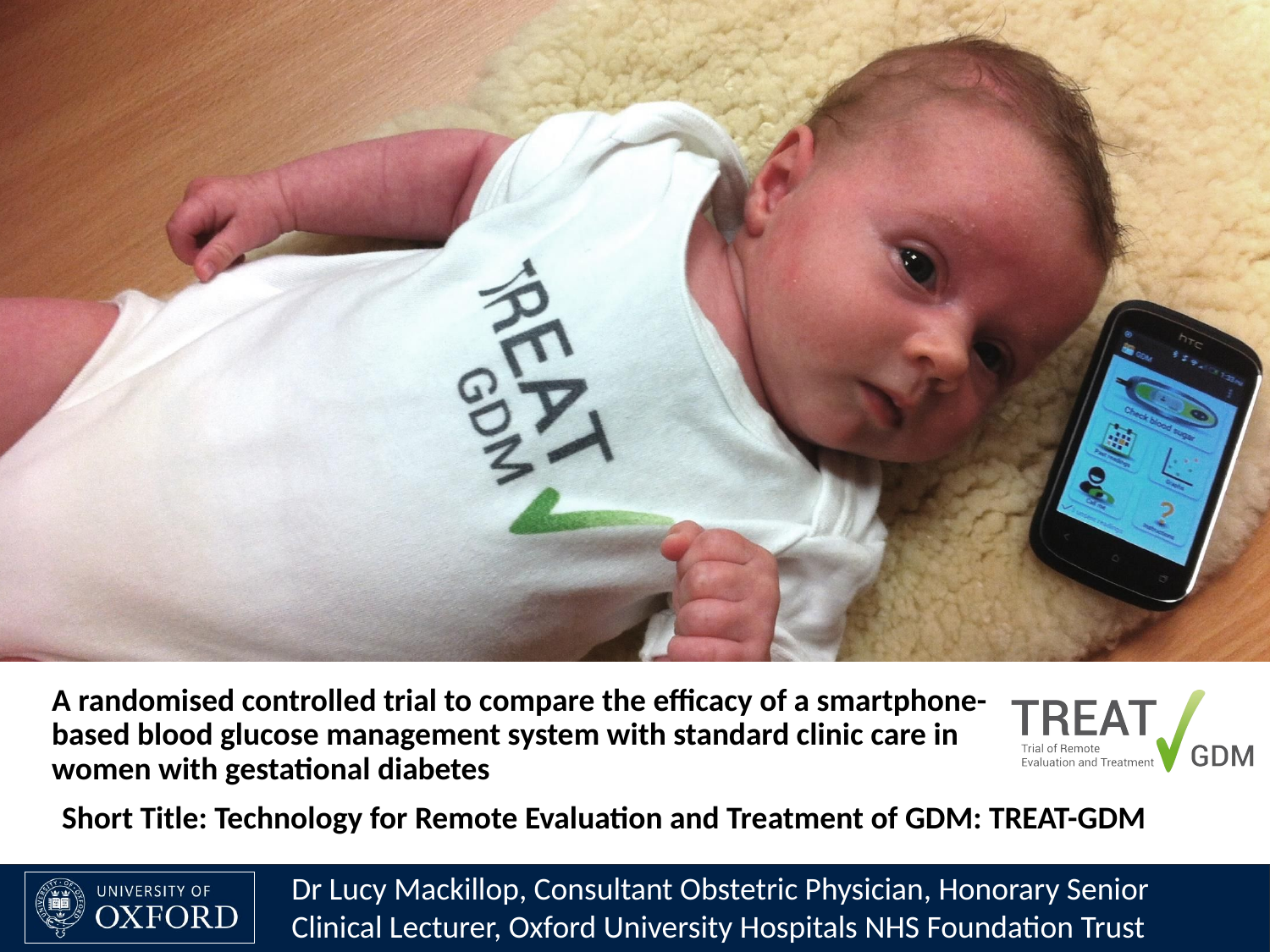

A randomised controlled trial to compare the efficacy of a smartphone-based blood glucose management system with standard clinic care in women with gestational diabetes
Short Title: Technology for Remote Evaluation and Treatment of GDM: TREAT-GDM
Dr Lucy Mackillop, Consultant Obstetric Physician, Honorary Senior Clinical Lecturer, Oxford University Hospitals NHS Foundation Trust

## Slide 2
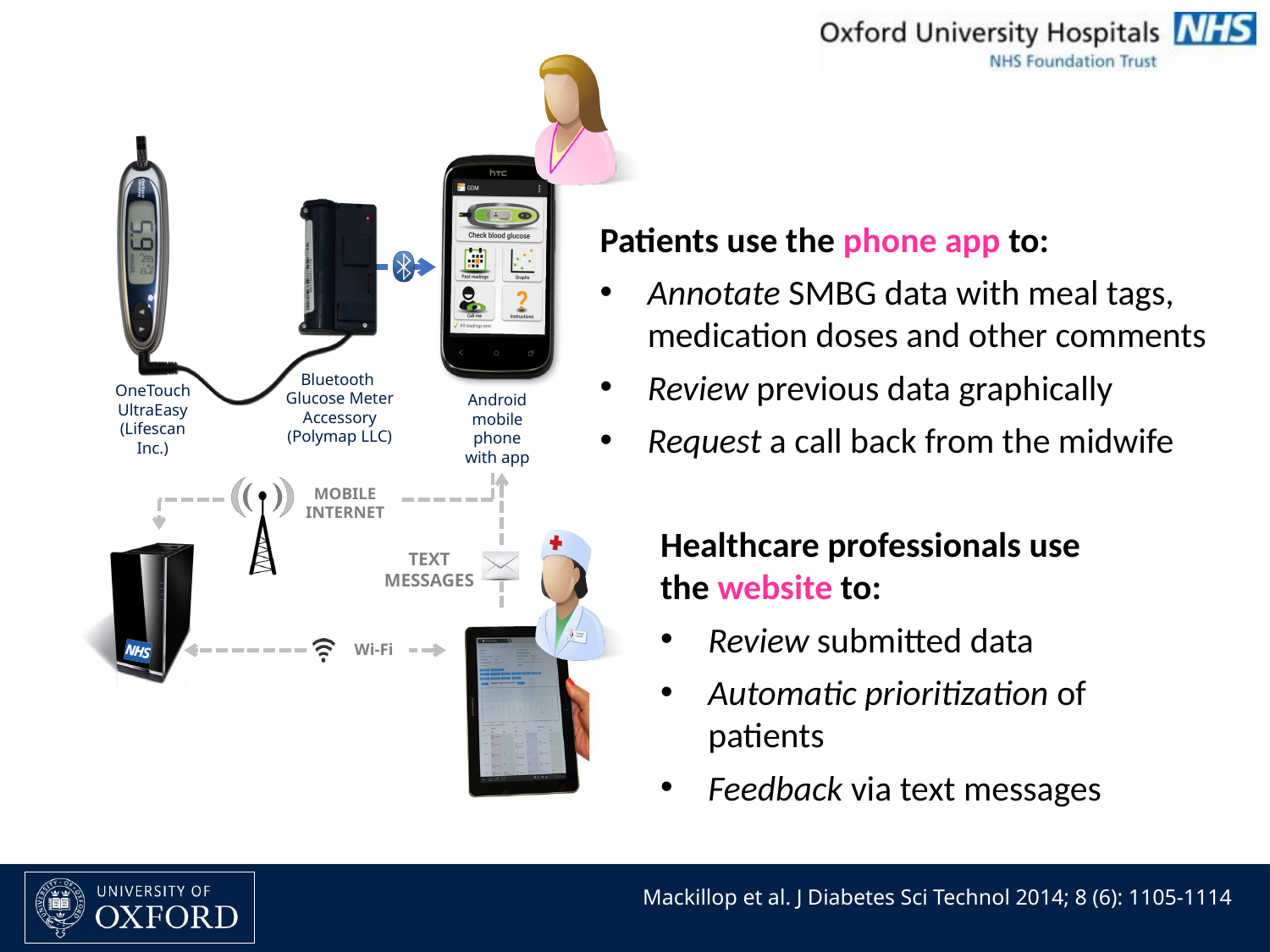

Bluetooth
Glucose Meter Accessory
(Polymap LLC)
OneTouch UltraEasy
(Lifescan Inc.)
Android mobile phone with app
MOBILE
INTERNET
TEXT
MESSAGES
Wi-Fi
Patients use the phone app to:
Annotate SMBG data with meal tags, medication doses and other comments
Review previous data graphically
Request a call back from the midwife
Healthcare professionals use the website to:
Review submitted data
Automatic prioritization of patients
Feedback via text messages
Mackillop et al. J Diabetes Sci Technol 2014; 8 (6): 1105-1114

## Slide 3
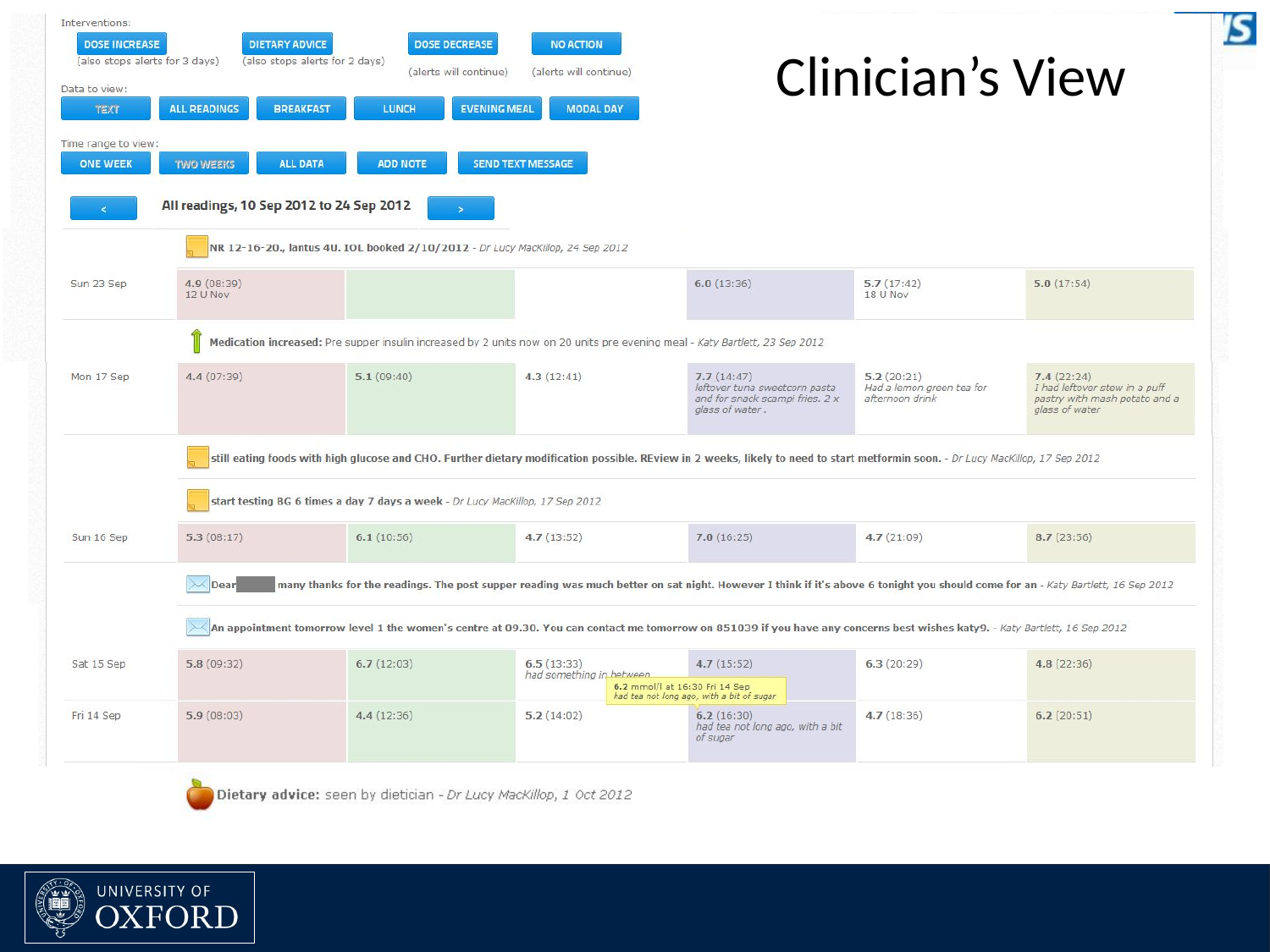

Clinician’s View

## Slide 4
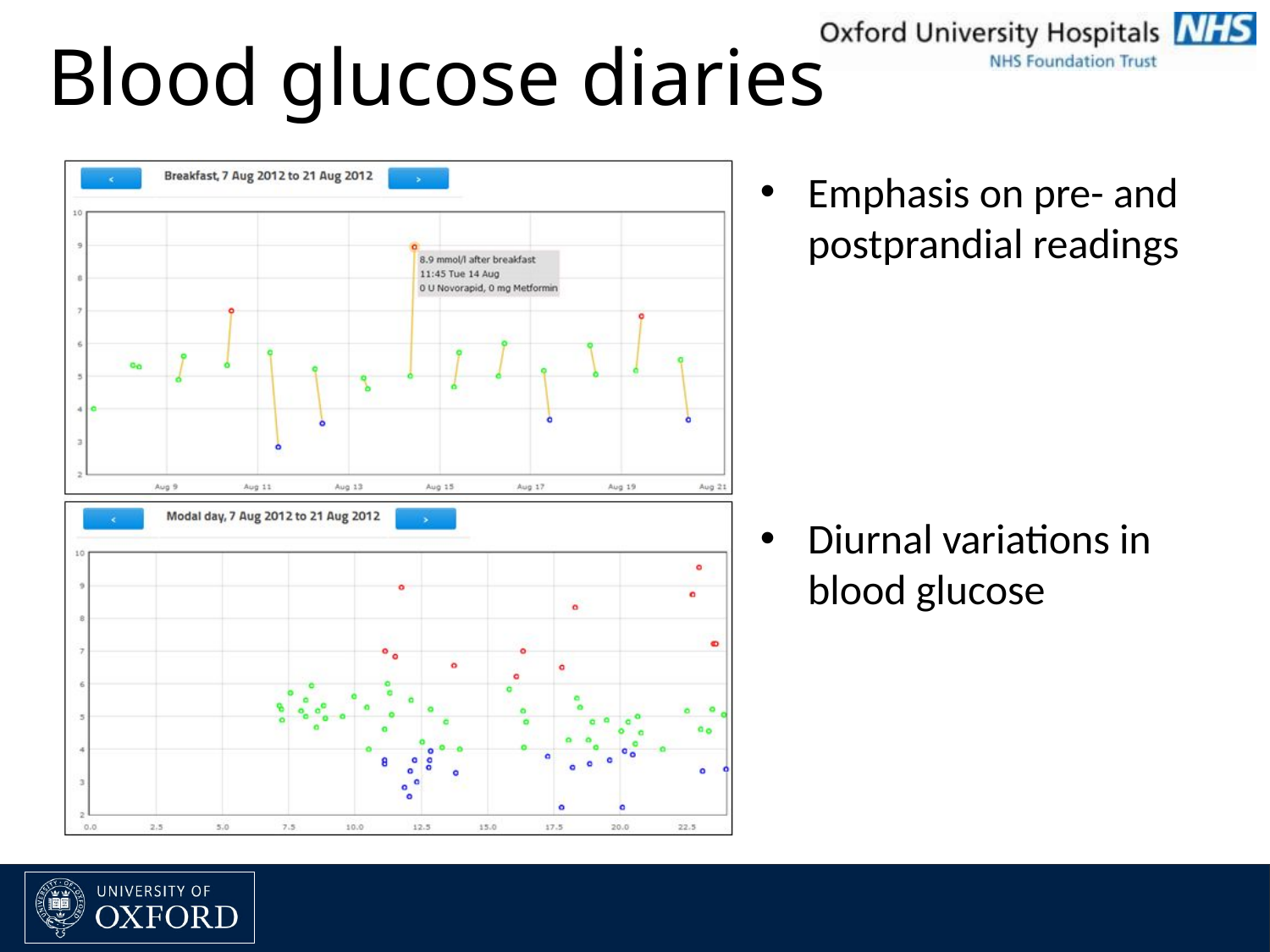

# Blood glucose diaries
Emphasis on pre- and postprandial readings
Diurnal variations in blood glucose

## Slide 5
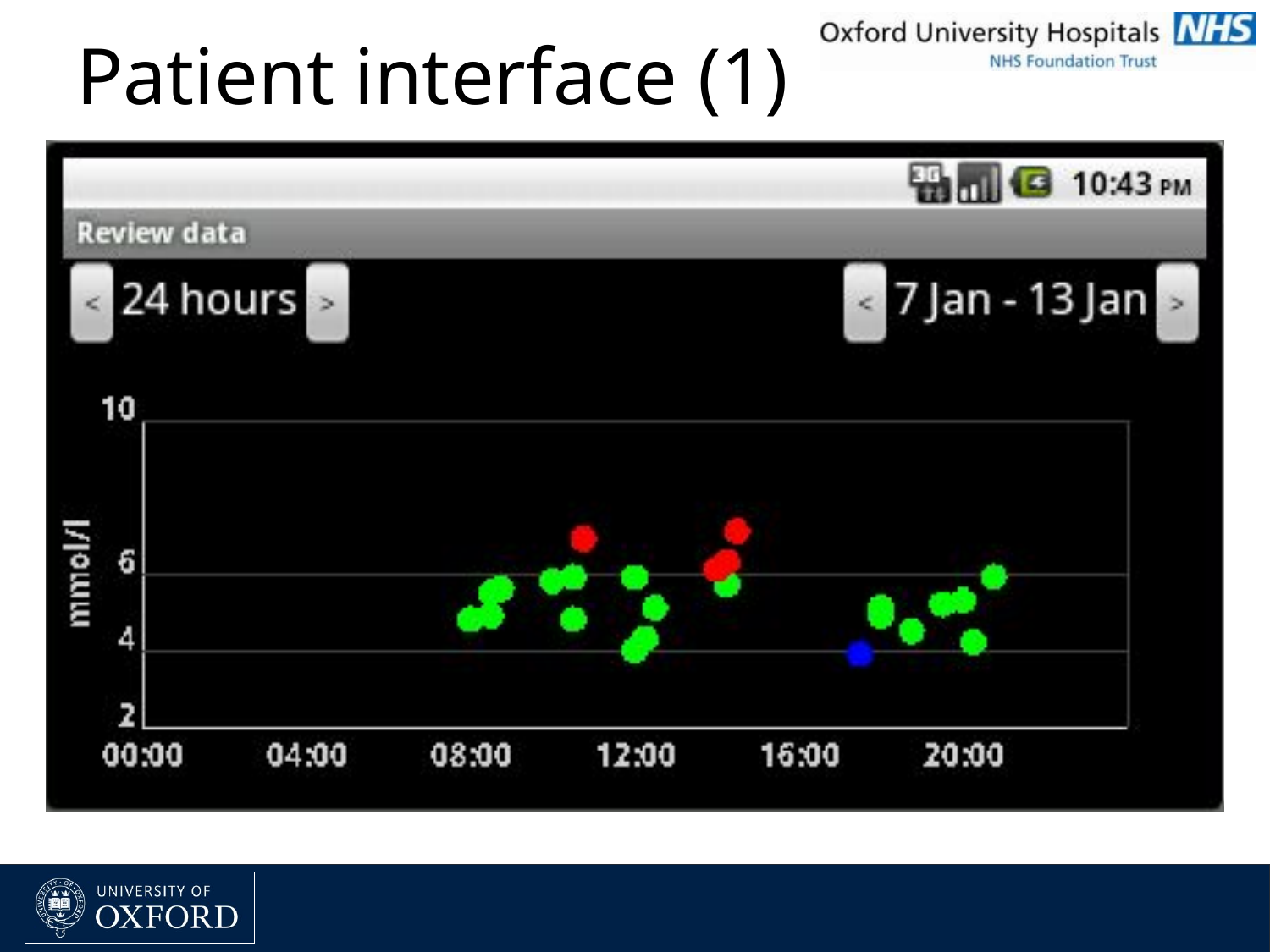

# Patient interface (1)

## Slide 6
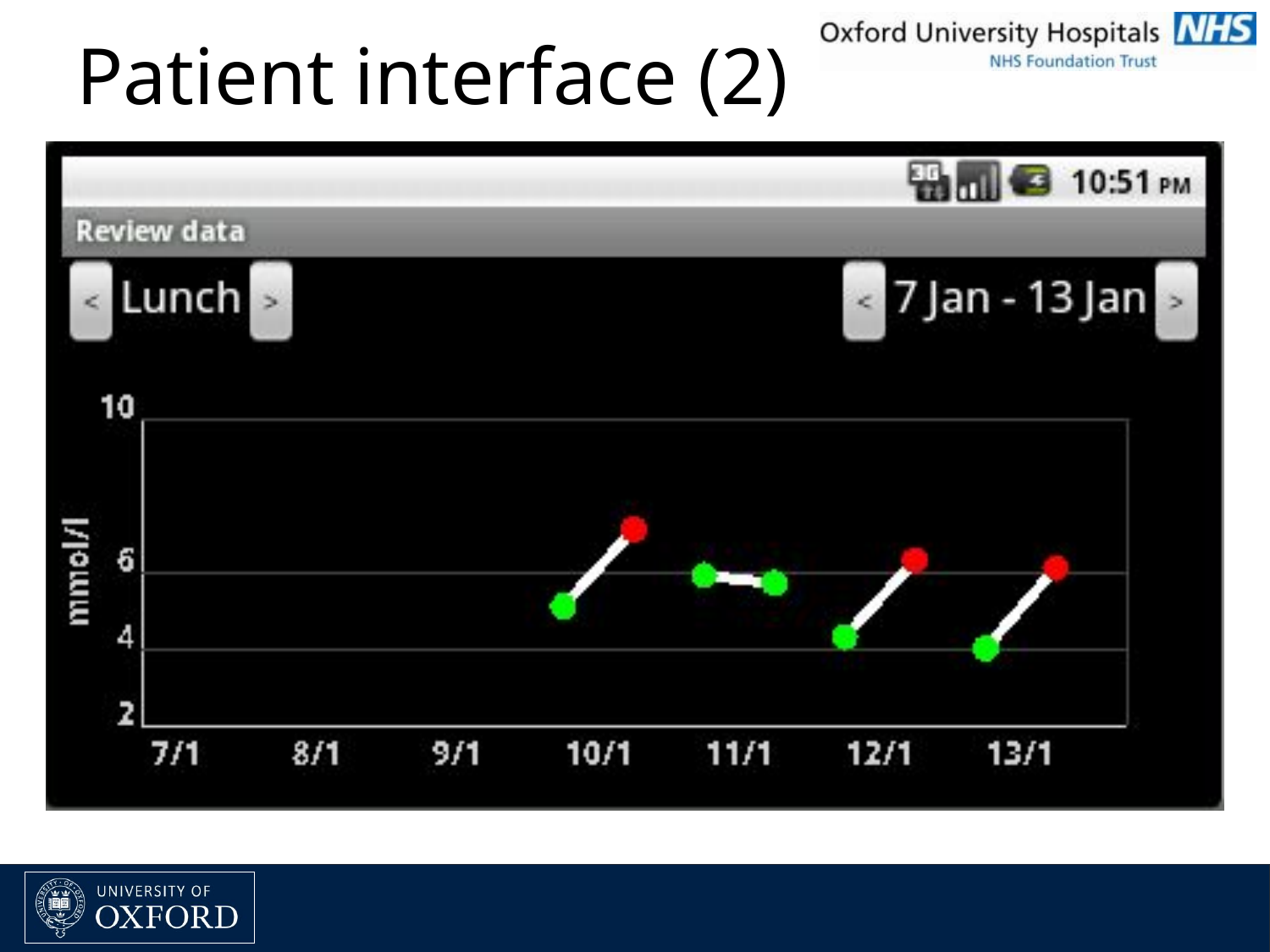

# Patient interface (2)

## Slide 7
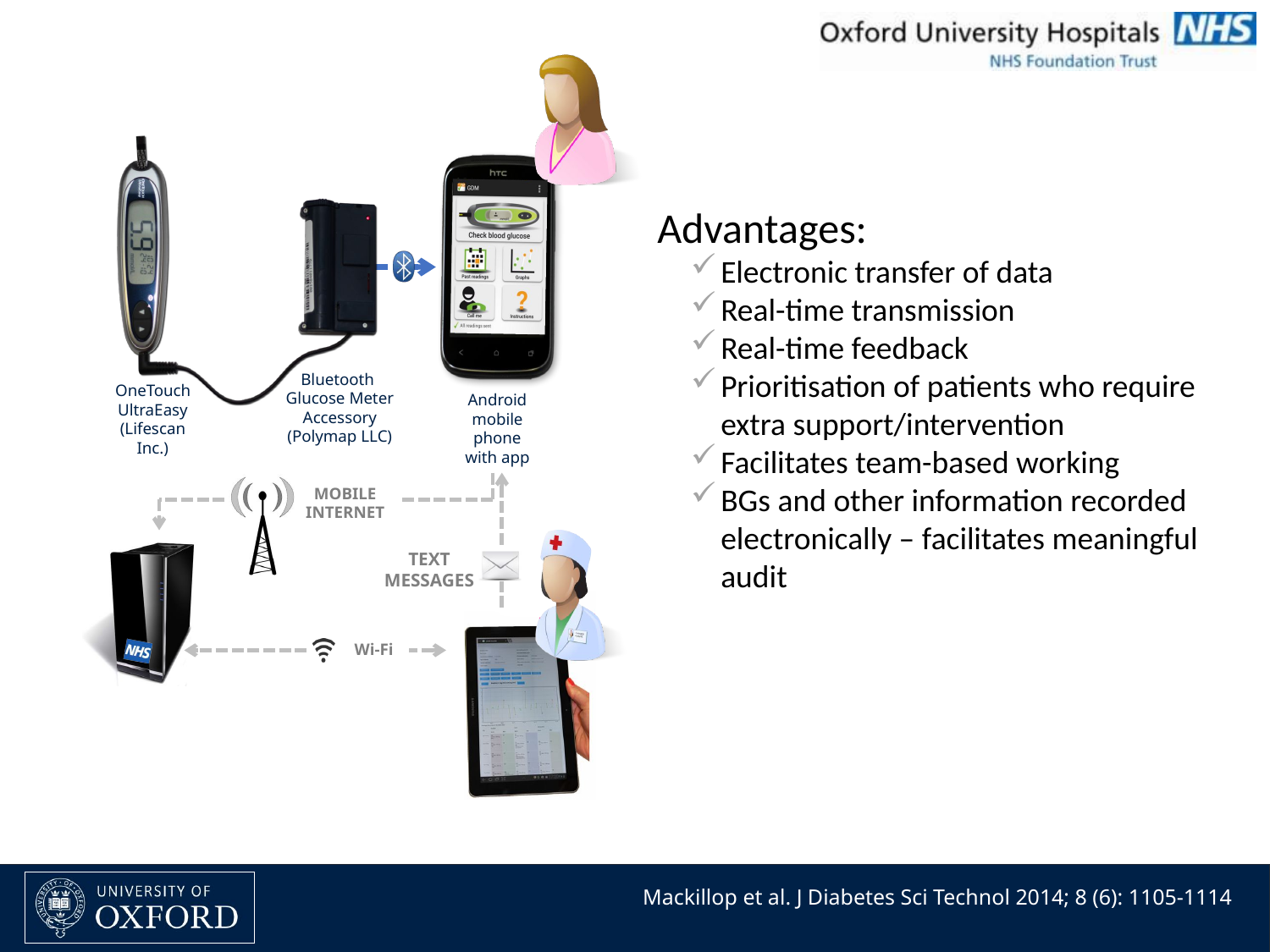

Bluetooth
Glucose Meter Accessory
(Polymap LLC)
OneTouch UltraEasy
(Lifescan Inc.)
Android mobile phone with app
MOBILE
INTERNET
TEXT
MESSAGES
Wi-Fi
Advantages:
Electronic transfer of data
Real-time transmission
Real-time feedback
Prioritisation of patients who require extra support/intervention
Facilitates team-based working
BGs and other information recorded electronically – facilitates meaningful audit
Mackillop et al. J Diabetes Sci Technol 2014; 8 (6): 1105-1114
