# Supplementary material for: Comparing the Efficacy of a Mobile Phone-Based Blood Glucose Management System With Standard Clinic Care in Women With Gestational Diabetes: Randomized Controlled Trial
Source: JMIR Mhealth Uhealth. 2018 Mar 20;6(3):e71. doi: 10.2196/mhealth.9512 (PMC5883074; doi:10.2196/mhealth.9512)
Supplement: Multimedia Appendix 2 [file mhealth_v6i3e71_app2.pdf]

Supplementary Information

Table A: Unit costs expressed in 2014/2015 UK pounds sterling prices used in the cost-analysis

Resource use variable	Unit cost (£)	Sources
**Antenatal care**		
**Community professional**		
General practitioner	£67	PSSRU
Community midwife	£56	NHS Reference Cost
**Secondary care**		
Hospital doctor (obstetrics clinic)	£124	NHS Reference Cost
Hospital midwife	£75	NHS Reference Cost
Dietitian	£83	NHS Reference Cost
Maternity day assessment unit	£395	NHS Reference Cost
**Intrapartum and postnatal care before discharge**		
Normal Delivery	£1,728	NHS Reference Cost
Assisted Delivery	£2,091	NHS Reference Cost
Planned Caesarean Section	£3,008	NHS Reference Cost
Emergency Caesarean Section	£3,838	NHS Reference Cost
3rd degree perineal trauma repair	£649	Birthplace cost-effectiveness analysis
Postpartum haemorrhage (>500 mL)	£1,201	NHS Reference Cost
Maternal stay (per night)	£103	NHS Reference Cost
Neonatal stay (per night)	£414	NHS Reference Cost
Neonatal special care baby unit stay (per night)	£486	NHS Reference Cost

Table B. Descriptive statistics for the blood glucose observations

	**Number of observations, number of patients, overall mean, range**	
	**intervention**	**control**
fasting	3850, 98, 5.26 (2.33-16.78)	2615, 85, 5.18 (1.70-12.00)
postprandial	9753, 98, 6.95 (2.36-23.61)	7090, 85, 6.90 (1.30-22.30)
preprandial	6479, 98, 5.09 (1.83-20.78)	4685, 85, 5.11 (1.30-11.70)
Highest weekly mean	6.86	6.18
Weeks after randomisation of occurrence of highest weekly mean	20	1

On target (fasting readings as defined ≥3.5 and ≤5.8 mmol/L and postprandial readings ≥3.5 and ≤7.7 mmol/L)

Table C. Secondary outcomes for blood glucose

Intervention			Control			Odds Ratio (95% CI)	p-value
**N women**	**N observations**	**N observations on target**	**N women**	**N observations**	**N observations on target**		
% of blood glucose fasting observations on target within 4 weeks of randomisation							
97	1934	1474	82	1486	1171	0.78 (0.45, 1.35)	p=0.37
% of blood glucose postprandial observations on target within 4 weeks of randomisation							
97	5006	3599	82	3975	2999	0.91 (0.67, 1.24)	p=0.55
% of blood glucose fasting observations on target between 4 and 8 weeks of randomisation							
89	1313	1089	70	884	734	0.75 (0.38, 1.48)	p=0.40
% of blood glucose postprandial observations on target between 4 and 8 weeks of randomisation							
87	3279	2473	70	2390	1908	0.69 (0.49, 0.99)	p=0.045

Results are reported from the generalised mixed linear logistic model adjusted for BMI at recruitment which is included as the only statistically significant fixed effectOn target (fasting readings as defined ≥3.5 and ≤5.8 mmol/L and postprandial readings ≥3.5 and ≤7.7 mmol/L)

Table D: mixed model analysis of blood glucose level

	Model 1		Model 2	
**Number of observations**	34269		34269	
**parameter**	Estimate (se)	p-value	Estimate (se)	p-value
**Fixed effects**				
**constant**	6.06 (0.32)		6.51 (0.37)	
**gestation (days)**	-0.0056 (0.0011)	p<0.0001	-0.0054 (0.0008)	p<0.0001
**group control**	-0.10 (0.42)	p=0.81	0.01 (0.07)	p=0.89
**treatment**	0		0	
**gestation x treatment**	0.0005 (0.0017)	p=0.79	-	
**meal breakfast**	0.075 (0.015	p<0.0001	0.075 (0.015	p<0.0001
**lunch**	-0.238 (0.016)	p<0.0001	-0.238 (0.016)	p<0.0001
**dinner**	0		0	
**post meal**	1.791 (0.012)	p<0.0001	1.791 (0.012)	p<0.0001
**pre meal**	0		0	
**smoking yes**	0.50 (0.20)	p=0.02	0.50 (0.20)	p=0.02
**no**	0		0	
**BMI at booking (kg/m^2)**	0.015 (0.005)	p=0.005	0.015 (0.005)	p=0.005
**Random effects**				
**Variance between intercepts**	4.333			
**Variance between gradients**	0.00007			
**Residual variance**	1.328			
**-2 Residual Log Likelihood**	107810			

The methods of linear mixed models were used to analyse these data. The dependent variable, blood glucose measurements, were taken by each patient six times per day between recruitment and delivery. The change in blood glucose over gestation was modelled using a linear regression equation. A random coefficient model was fitted which allowed for differences between patients in the rate of change of blood glucose. To model the correlation over gestation within patients the unstructured covariance matrix was used. A two-level factor indicating the treatment group, a factor with three levels indicating the time of day of the blood glucose measurement, breakfast, lunch or dinner, together with a two level factor, indicating whether the measurement was pre or post meal, were included in the model as fixed effects. To test whether there was a difference in the mean rate of change of blood glucose over gestation between the treatment groups, the interaction between treatment group and gestation was included as a fixed effect (model 1). The interaction was not significant and was dropped from a second analysis (model 2). Baseline characteristics were included as fixed effects, and these may explain some of the variation between patients in the rate of change of blood glucose over gestation.
