# Supplementary material for: Comparing the Efficacy of a Mobile Phone-Based Blood Glucose Management System With Standard Clinic Care in Women With Gestational Diabetes: Randomized Controlled Trial
Source: JMIR Mhealth Uhealth. 2018 Mar 20;6(3):e71. doi: 10.2196/mhealth.9512 (PMC5883074; doi:10.2196/mhealth.9512)
Supplement: Multimedia Appendix 3 [file mhealth_v6i3e71_app3.pdf]

Outcome	Intervention		Control		Difference Intervention vs control	
	N	n (%) or mean (SD)	N	n (%) or mean (SD)	OR or mean difference (95% CI)	P value
Gestational age at delivery in weeks[1]	101	39.1 (38.8-39.7)	102	38.7 (38.3-39.4)	logrank test chisq(1) =1.48	p=0.22
Mode of delivery	101		102		X^2_3= 14.5	p=0.005
Vaginal		52 (51.4)		42 (41.2)		
Caesarean elective		21 (20.8)		23 (22.5)		
Caesarean emergency		6 (5.9)		24 (23.5)		
Assisted		22 (21.8)		13 (12.7)		
Major perineal trauma[a]	80	3 (4.0)	78	1 (0)	3.03 (0.31, 29.6)	p=0.34
Weight gain between recruitment and delivery (kg/week)	96	1.23 (0.96)1	98	1.19 (1.20)	0.04 (-0.26, 0.35)	p=0.77
Maternal pregnancy-induced hypertension or preeclampsia	101	1 (1.0)	102	5 (4.9%)	0.20 (0.004, 1.79)	p=0.22
Admission to higher level of care for mother	100	01	99	01	n/a	
Hypoglycaemic medication at delivery[b]	84	28 (33)	89	44 (49)	0.63 (0.36, 1.10)	p=0.11
Delivery less than 37^{+0} weeks	101	5 (5.0)	102	13 (12.7%)	0.36 (0.12, 1.04)	p=0.064
Birth weight in grams (sd)	101	3440 (516)	101	3338 (559)	102 (-47, 251)	p=0.18
Birth weight centile (median, IQR)	100	75.2 (47.1, 90.6)	99	76.4 (47.1,87.2)	Kruskal Wallis test chisq(1)= 0.33	p=0.57
Birth weight centile > 90[c]	100	26 (26.0)	99	19 (19.2)	1.48 (0.76, 2.89)	p=0.25
Sex (female)	101	50 (49.5%)	102	47 (46.1%)	1.15 (0.66, 1.99)	p=0.62
Shoulder dystocia	100	1 (1.0)	102	0 (0)	1.02 (.026, inf)	p=0.99
Neonatal hypoglycaemia	96	31 (32.3)	93	25 (26.9)	1.30 (0.69, 2.43)	p=0.42
Neonatal jaundice	100	10 (10.0)	95	7 (7.4)	1.38 (0.50, 3.79)	p=0.53
Admission to higher level of care for baby	101	5 (5.0)	99	12 (12.1)	0.38 (0.13, 1.12)	p=0.08

[a]*conditional on not having an elective caesarean section*
[b]*conditional on not taking medication at recruitment*
[c]*missing values when gestational age at delivery<33 weeks*
