# Supplementary material for: Comparing the Efficacy of a Mobile Phone-Based Blood Glucose Management System With Standard Clinic Care in Women With Gestational Diabetes: Randomized Controlled Trial
Source: JMIR Mhealth Uhealth. 2018 Mar 20;6(3):e71. doi: 10.2196/mhealth.9512 (PMC5883074; doi:10.2196/mhealth.9512)
Supplement: Multimedia Appendix 4 [file mhealth_v6i3e71_app4.pdf]

Breakdown of healthcare resource use and associated cost of intervention and control groups. Costs expressed in 2014/2015 UK pounds sterling prices. Values represent arithmetic mean (standard deviation) per delivery unless stated otherwise.

**Healthcare resource use category**	**Intervention Group (n=101)**					**Control Group (n=102)**					**Parametric cost difference and 95% CI**
	**n**	**Min**	**Max**	**Mean resource use (SD)**	**Mean Cost (SD)**	**n**	**Min**	**Max**	**Mean resource use (SD)**	**Mean Cost (SD)**	
					**Antenatal care**						
**Community**											
General practice	93	0	4	0.51 (0.76)	£34 (£51)	97	0	3	0.51 (0.75)	£34 (£50)	£0 (-£14 to £15)
Community midwife	93	0	7	2.18 (1.40)	£122 (£78)	97	0	8	1.89 (1.64)	£106 (£92)	£17 (-£7 to £41)
**Secondary care**											
Hospital doctor	93	0	12	4.65 (2.89)	£576 (£358)	97	0	13	5.06 (2.86)	£628 (£356)	-£52 (-£154 to £50)
Hospital midwife	93	1	14	3.94 (2.40)	£295 (£180)	97	0	11	3.95 (2.14)	£296 (£160)	-£1 (-£50 to £48)
Dietician	93	0	1	0.13 (0.34)	£11 (£28)	97	0	1	0.23 (0.42)	£19 (£35)	-£8 (-£17 to £1)
Maternity day assessment unit	93	0	9	1.17 (1.74)	£463 (£686)	97	0	10	1.44 (1.83)	£570 (£723)	-£107 (-£309 to £95)
**Total mean cost antenatal care (1)**	**93**				**£1,501 (£889)**	**97**				**£1,652 (£950)**	**-£151 (-£415 to £112)**
					**Intrapartum and postnatal care before discharge**						
Mode of birth (n, %)											
Vaginal	101			52 (0.52)	£890 (£868)	102			42 (0.41)	£712 (£855)	£178 (-£60 to £417)
Assisted delivery	101			22 (0.22)	£456 (£867)	102			13 (0.13)	£267 (£701)	£189 (-£29 to £407)
Caesarean elective	101			21 (0.21)	£625 (£1,227)	102			23 (0.23)	£678 (£1,263)	£53 (-£397 to £291)
Caesarean **emergency**	101			6 (0.06)	£228 (£912)	102			24 (0.24)	£903 (£1,636)	-£675 (-£1042 to -£291)a

Breakdown of healthcare resource use and associated cost of intervention and control groups. Costs expressed in 2014/2015 UK pounds sterling prices. Values represent arithmetic mean (standard deviation) per delivery unless stated otherwise.

Postpartum haemorrhage (>500 mL) (n,%)	100			42 (0.42)	£504 (£596)	101			50 (0.50)	£595 (£603)	-£90 (-£257 to £77)
3rd degree perineal trauma repair (n,%)	101			3 (0.03)	£19 (£110)	100			0 (0.00)	£0	£19 (-£3 to £41)
Hospital length of stay											
Duration of maternal stay from delivery	101	0	14	2.38 (1.96)	£245 (£202)	99	0	15	2.89 (2.43)	£298 (£250)	-£53 (-£116 to £11)
Duration of neonatal stay	101	0	50	2.93 (5.23)	£1,213 (£2,164)	99	0	89	4.23 (9.87)	£1752 (£4,086)	-£539 (-£1,448 to £370)
Duration of special care baby unit stay	101	0	1	0.05 (0.22)	£24 (£106)	99	0	1	0.12 (0.33)	£59 (£159)	-£35 (-£73 to £3)
**Total mean cost intrapartum and postnatal care before discharge (2)**	**100**				**£4,219 (£2,733)**	**97**				**£5,208 (£4,507)**	**-£989 (-£2,033 to £55)**
**Total mean care cost per delivery (1) + (2)**	**92**				**£5,697 (£3,068)**	**93**				**£6,741 (£4,640)**	**-£1,044 (-£2,186 to £99)**

SD: standard deviation; CI: confidence interval; [a]p=0.0004
